# UPLC/ESI/MS profiling of red algae *Galaxaura rugosa* extracts and its activity against malaria mosquito vector, *Anopheles pharoensis*, with reference to *Danio rerio* and *Daphnia magna* as bioindicators

**DOI:** 10.1186/s12936-023-04795-w

**Published:** 2023-12-01

**Authors:** Mohamed A. M. El-Tabakh, Esraa A. Elhawary, Hossam M. Hwihy, Kareem F. Darweesh, Raafat M. Shaapan, Emad A. Ghazala, Mostafa M. Mokhtar, Hassan O. Waheeb, Deyaa E. M. Emam, Nader A. Bakr, Ahmed Z. I. Shehata

**Affiliations:** 1 Zoology Department, Faculty of Science, Al-Azhar University, Cairo, 11651 Egypt; 2 Department of Pharmacognosy, Faculty of Pharmacy, Ain-Shams University, Cairo, Egypt; 3 Department of Zoonosis, Veterinary Research Institute, National Research Centre, Giza, Egypt; 4 EEAA, Ras Muhammed National Park, Qesm Sharm Ash Sheikh, South Sina Egypt

**Keywords:** *Galaxaura rugosa*, *Anopheles pharoensis*, Extract, Larvicidal, LCMS, Metabolomics

## Abstract

**Background:**

*Anopheles pharoensis* has a major role in transmitting several human diseases, especially malaria, in Egypt?. Controlling *Anopheles* is considered as an effective strategy to eliminate the spread of malaria worldwide. *Galaxaura rugosa* is a species of red algae found in tropical to subtropical marine environments. The presence of *G. rugosa* is indicative of the ecosystem's overall health. The current work aims to investigate UPLC/ESI/MS profile of *G. rugosa* methanol and petroleum ether extracts and its activity against *An. pharoensis* and non-target organisms, *Danio rerio* and *Daphnia magna*.

**Methods:**

*Galaxaura rugosa* specimens have been identified using DNA barcoding for the COI gene and verified as *G. rugosa.* The UPLC/ESI/MS profiling of *G. rugosa* collected from Egypt was described. The larvicidal and repellent activities of *G. rugosa* methanol and petroleum ether extracts against *An. pharoensis* were evaluated, as well as the toxicity of tested extracts on non-target organisms, *Dan. rerio* and *Dap. magna.*

**Results:**

The UPLC/ESI/MS analysis of methanol and petroleum ether extracts led to the tentative identification of 57 compounds belonging to different phytochemical classes, including flavonoids, tannins, phenolic acids, phenyl propanoids*.* Larval mortality was recorded at 93.33% and 90.67% at 80 and 35 ppm of methanol and petroleum ether extracts, respectively, while pupal mortality recorded 44.44 and 22.48% at 35 and 30 ppm, respectively. Larval duration was recorded at 5.31 and 5.64 days by methanol and petroleum ether extracts at 80 and 35 ppm, respectively. A decrease in acetylcholinesterase (AChE) level and a promotion in Glutathione-S-transferase (GST) level of *An. pharoensis* 3rd instar larvae were recorded by tested extracts. The petroleum ether extract was more effective against* An. pharoensis* starved females than methanol extract. Also, tested extracts recorded LC_50_ of 1988.8, 1365.1, and 11.65, 14.36 µg/mL against *Dan. rerio,* and *Dap. magna,* respectively.

**Conclusions:**

Using red algae derivatives in *An. pharoensis* control could reduce costs and environmental impact and be harmless to humans and other non-target organisms.

**Supplementary Information:**

The online version contains supplementary material available at 10.1186/s12936-023-04795-w.

## Background

Marine red algae are a diverse group of seaweeds often found on rocks or dead coral pieces in the upper subtidal zone of the Atlantic, Indian, and Pacific Oceans, where they are exposed to moderate wave action [1]. The seaweed *Galaxaura rugosa* has just been identified on the coasts of South Africa but is most usually found in the waters of Japan, Korea, Taiwan, Vietnam, Singapore, Indonesia, the Philippines, Australia, New Zealand, and the Pacific Islands [2–6]. The algaeof the genus *Galaxaura* produce various bioactive compounds, such as sulfated polysaccharides, phycobiliproteins, fatty acids, and other secondary metabolites. Bioactive chemicals with antioxidant, antiviral, antifungal, and antibacterial properties have been isolated from the red marine alga *Galaxaura elongata* [1, 7].

Mosquitoes, especially *Anopheles* genera because of their role in transmitting several animal and human diseases, such as malaria [8]. Malaria is the world's most widespread parasitic disease, caused by *Plasmodium* protozoa, which has infected about 241 million people and caused 627,000 deaths worldwide, in 2021 [9]. Several strategies have been applied to control the prevalence of *Anopheles* spp. and thus eliminate the spread of malaria [10]. Chemical insecticides have usually targeted aquatic larvae of different *Anopheles* spp. for many years; however, developing new control agents, which are more safe, efficient, and eco-friendly, considered a proper and necessary replacement to avoid the hazards of chemical insecticides [11, 12]. Red marine algae bioactive compounds have been shown to have insecticidal properties against different pests, such as mosquitoes, flies, aphids, and caterpillars [13].

The Zebrafish *Danio rerio* has many advantages as a toxicologic model in view of its easy maintenance, fast maturation, and successful laboratory acclimation [14]. *Daphnia magna* is a freshwater crustacean species belonging to the *Daphnia* genus*.* Both the zebrafish and *Daphnia* are used as non-target model organisms in ecology and evolution, a bioindicator of water quality, and a test organism for ecotoxicology [15]. Specifically, in the context of this study, *Daphnia* and zebrafish were used to assess the potential off-target effects of *G. rugosa* extracts, ensuring that these agents, while lethal to *Anopheles pharoensis*, were not indiscriminately harmful to non-target organisms. Their use provides a comprehensive understanding of the insecticidal potential of *G. rugosa,* as well as its broader ecological impact [15].

## Methods

### Ethical approval

This study was performed in Animal House, Zoology Department, Faculty of Science, Al-Azhar University, Cairo, Egypt, according to ethics of Zoology Department, Faculty of Science, Al-Azhar University.

### Collection and preparation of algae extracts

#### Site of sampling

**Fig. 1:**
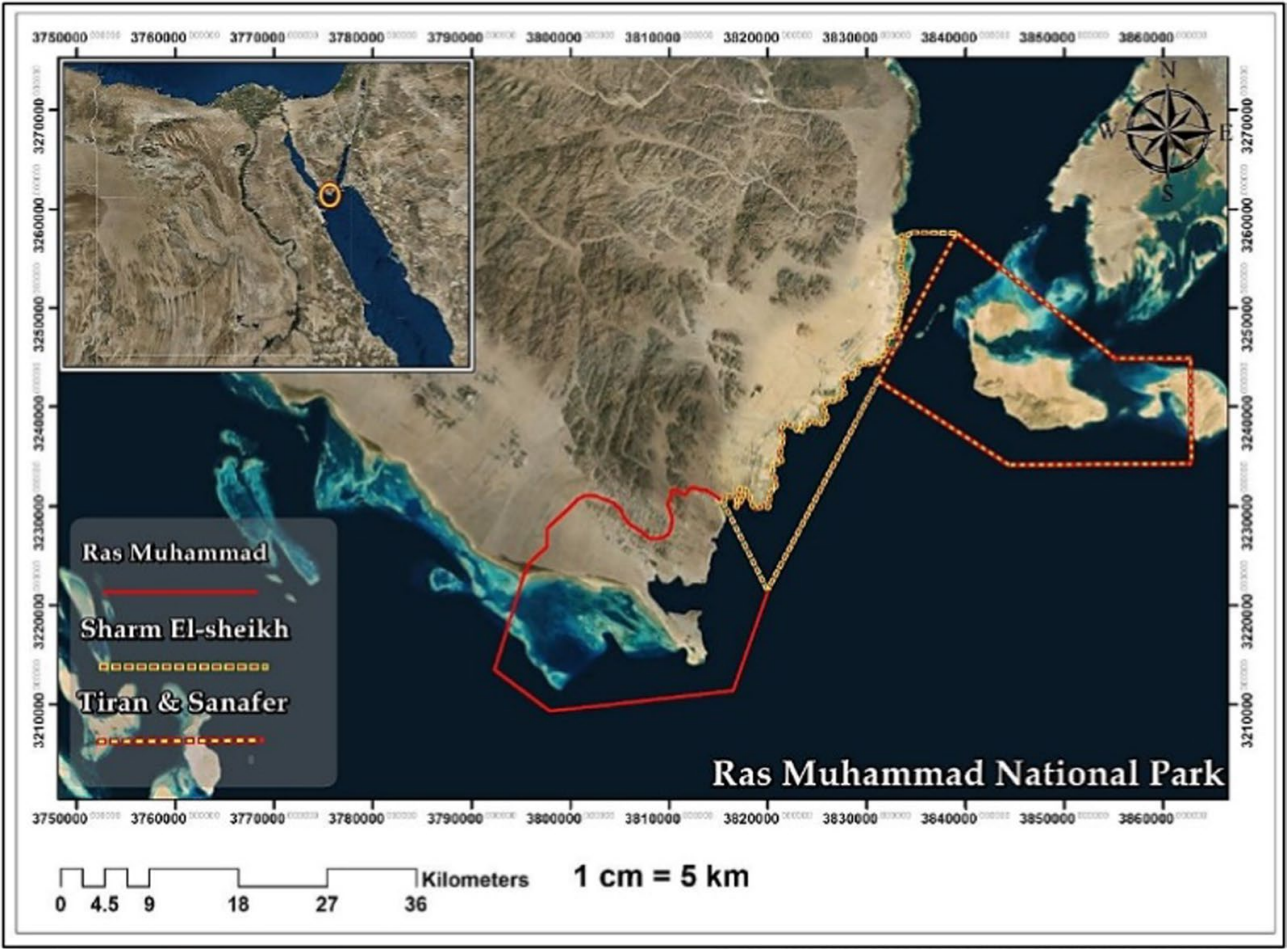
The boundaries of Ras Muhamad National Park

**Fig. 2:**
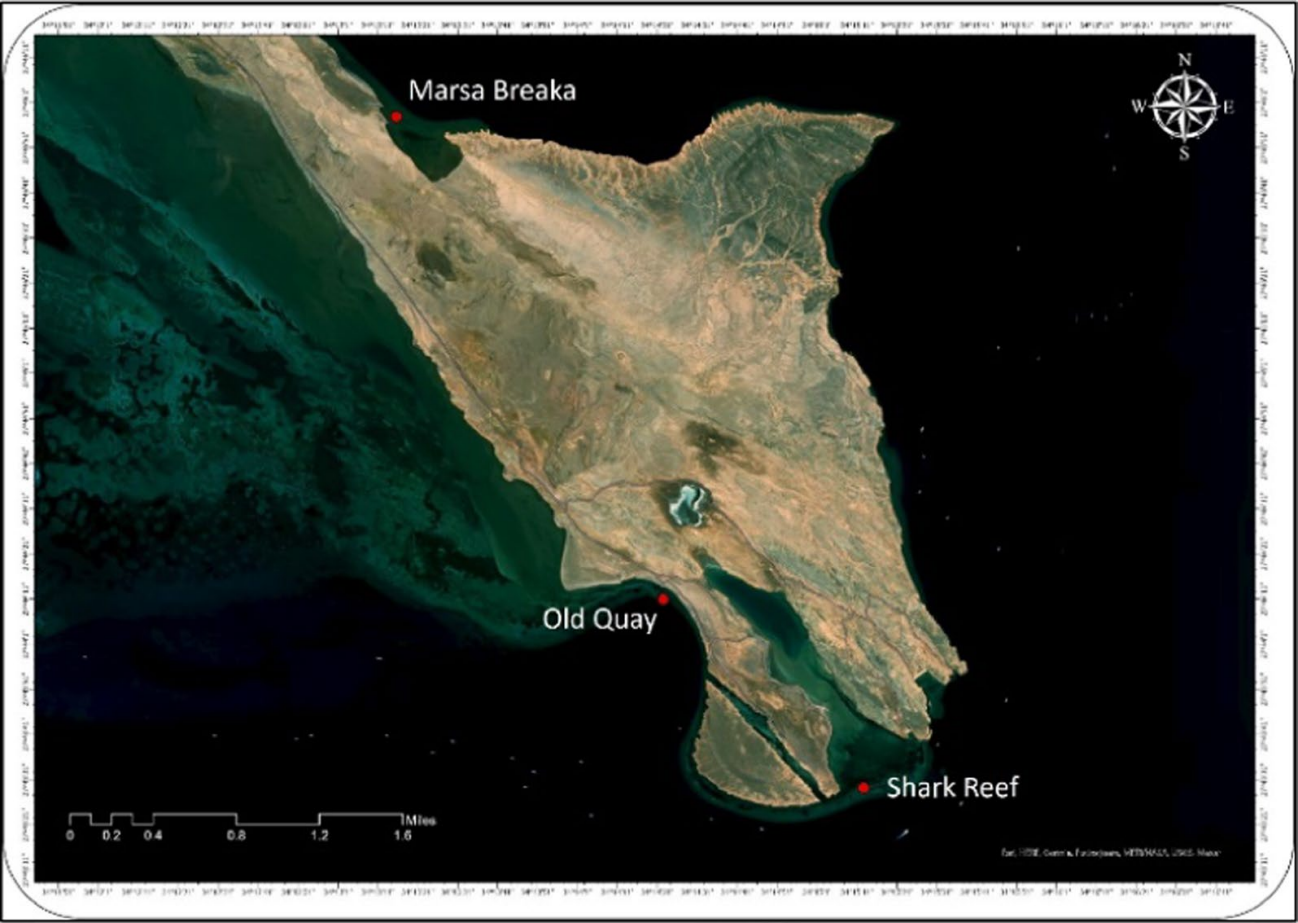
Location of sampling sites

The sampling of *G. rugosa* was mainly conducted at Ras Muhammad National Park, located at 27°43′20″N & 34°15′14″E at three different sites distinguished by the habitats. The 1st

site was Shark Reef, 2nd site was Old Quay, and 3rd site was Marsa Breaka (Figs. 1 and 2).

### Field sampling and identification

The seaweed samples were collected in April 2023. Sampling was done by snorkeling and scuba diving, and specimens were preserved in frozen seawater. A Garmin GPS device was used to determine the coordinates of the sampling sites. The marine biology section of the Zoology Department of the Faculty of Science et al.-Azhar University in Cairo, Egypt, used the procedures described in the AlgaeBase website to confirm the identification of the samples [16, 17]. The present research sequenced samples because of the narrow gap between algae species, necessitating cutting-edge methods to ensure correct classification. The red algae DNA was extracted using a tweaked approach that allowed us to amplify the COX1 gene region [18]. The PCR amplification profile continued, but the annealing temperature decreased to 50 degrees Celsius [19]. Gel purification was used on amplified PCR products [20]. The PCR forward and reverse sequencing readings were edited and aligned in MEGA V14.0. Using the Basic Local Alignment Search Tool (BLAST) at http://blast.ncbi.nlm.nih.gov/Blast.cgi, The newly acquired COI sequences of *G. rugosa* (Accession number: OR362159-61) BankIt to those in GenBank.

### Preparation of the algae extractions

Air-drying *G. rugosa* took 2 days. It was then baked at 40ºC for 2–3 days, or until the weight stays the same. The dried biomass was ground up in a standard kitchen blender to get a powder. 100 g of fine material was extracted for further study [21]. Both methanol and petroleum ether (20 g) were used to extract the moisture-free seaweed material in a Soxhlet extractor at 40 °C for 7 h. After filtering the whole extract, the resulting crude extract was concentrated in a rotary evaporator at 40ºC until completely dry [22]. The obtained residue was transferred into 100 mL glass beakers and stored at 4 °C until used.

### Ultra performance liquid chromatography-electrospray ionization-mass spectrometry (UPLC/ESI/MS) analysis

Positive and negative ion acquisition modalities of UPLC-ESI–MS were performed according to the established protocol [23].

### Anopheles pharoensis colonization

*Anopheles pharoensis* larvae were collected from Faiyum Governorate, Egypt (29^º^18^´^53.4^''^ N, 30^º^39^´^19.2^''^ E, altitude 19 m) and identified using a previously described key [24]. Collected larvae were transferred into mosquito insectary, Animal House, Zoology Department, Faculty of Science, Al-Azhar University, Cairo, Egypt, under controlled conditions of temperature (25–27 °C), relative humidity (70–80%) and photoperiod (12L:12D). A standard rearing procedure followed to provide larvae needed for the bioassay [25].

### Larvicidal activity of the tested extracts

The previously described larvicidal bioassay procedure was applied with minor modifications [23]. The larvae were separated and placed in 350 ml beakers containing 250 ml of distilled water with 2 drops of Tween_80_ with varying amounts of the extracts being evaluated. In most cases, three sets of 25 third-instar larvae were employed. The α-Cypermethrin (produced by Sidasa Company, Cairo, Egypt, for fertilizers, pesticides, and chemicals) was employed as a positive control agent, and control larvae were treated with 2 drops of Tween_80_ in 250 ml distilled water.

### Enzymatic measurements

Acetylcholinesterase (AChE) plays a critical role in the termination of nerve impulse transmission at cholinergic synapses by hydrolyzing the neurotransmitter acetylcholine. While Glutathione S-transferase (GST) is an enzyme involved in the detoxification of xenobiotics and endogenous compounds by conjugating them with glutathione, aiding in their subsequent elimination from the organism. Lastly, Superoxide dismutase (SOD), is an essential antioxidant enzyme that defends cells against oxidative damage by catalyzing the dismutation of superoxide anions into hydrogen peroxide and molecular oxygen. In the context of present study, monitoring the activities of these enzymes provides insights into the physiological responses of the tested organisms to *G. rugosa* extracts, shedding light on potential modes of action and effects beyond mere mortality. The impact of the extracts was studied using half-lethal doses (LC_50_). For the measurement of AChE, GST, and SOD, 10 ml solutions of 0.1 M-phosphate buffer, pH 7.5 (KH_2_PO_4_ -NaOH), containing 1% Triton X-100, 1% Triton X-100, 1% ethanol, and 1% Triton X-100, respectively, were used to homogenize 3 batches of larvae (obtained from each tested LC_50_). Hereaeus Labofuge 400R, Kendro Laboratory Products GmbH, Germany, was used to centrifuge the homogenates for 60 min at 4 °C and 15.000 × g. The resultant supernatant was put through an AChE (U/L) inhibition experiment in vitro without further purification [26–29]. GST activity (U/g tissue) was determined by doing spectrophotometric measurements of aliquots of the supernatant in accordance with the protocol described in the accompanying pamphlet [30]. The SOD activity (U/mg tissue) was determined according to 2007 manufacturer’s instructions (R&D Systems, Inc.). Aliquots of 50 mL were collected from the supernatant for spectrophotometric analyses.

### Repellency test

The repellent activity of the tested extracts was examined using a procedure described with small modifications [10]. Fifty *An. pharoensis* starved females were kept in net cages (45 × 30 × 45 cm). Three doses of the tested extracts (6.67, 3.33, and 1.67 mg/cm^2^) were prepared in 2 ml methanol or petroleum ether with 2 drops of Tween_80_. Methanol and petroleum ether with 2 drops of Tween_80_ were used as controls. Positive control (DEET) was purchased from a commercial pharmacy. Three replicates were usually used along with the control. The repellency percentages were calculated using a standard formula [28].

### Toxicity to the non-target organisms

#### Zebrafish model

Established aquaria of the Laboratory of Fish Rearing at the Animal House of the Zoology Department in the Faculty of Science et al.-Azhar University in Cairo, Egypt, Zebrafish, *Danio rerio* reared for providing a stock. The Al-Azhar University Animal Research Ethics Committee's standards were followed in treating the test subjects (Egypt). They were acclimated in 1000-millilitre circular aquaria. Ten fish were kept in each tank, which was aerated artificially around the clock. The fish were given fish food that had the right size pellets for them. The tests were run in triplicate [29]. Thirty adults of healthy Zebrafish were subjected to various amounts of each investigated item for 96 h to get insight into the influence of these substances on present non-target model. Mortality was reported 96 h after therapy was given to the control group subjected to the same tests. The method of Deo et al*.* was used to calculate the estimated toxicity in terms of a percentage [30, 31].

### Daphnia magna model

*Daphnia magna* came from the invertebrate breeding facility. A yeast powder solution raised both nymphs and adults in 10-L water tanks. Total hardness ranged from 35 to 50 mg CaCO3 L^−1^, pH ranging from 7.15 to 7.5, the temperature was constant at 25 ± 1 °C, electrical conductivity was around 160 µS.cm^−1^, dissolved oxygen was about 4 mgl^−1^, and pH was 7.15–7.5 [31]. The acute toxicity tests were conducted mostly in accordance with the OECD recommendations (Test no. 202, *Daphnia* sp., Acute Immobilization Test) [32], but with the essential changes noted below. Twenty *Daphnia magna* neonates were subjected to each test tank, with a total of four repetitions. Individual *Daphnia* were tested for 48 h in containers containing 250 mL of clean water and various amounts of materials. A stereomicroscope was used to view the organisms at the conclusion of the acute toxicity tests, and the number of dead neonates in each of the four replicates was used to calculate the LC_50_ after 48 h. The individual was considered dead if the stereomicroscope revealed no signs of life [33]*.*

### Statistical analysis

Mean ± SD was how the data were presented. ANOVA was used to evaluate the data, as recommended [34]. SPSS V.22 was used for data encoding and entry. Quantitative data were reported using mean, and standard deviation; qualitative data were presented with frequency. The threshold for statistical significance was set at P < 0.05. All stations polled throughout the research period had their parameters' correlation coefficients calculated using the computer application MINITAB V.14. With R-studio 4.1.3, data was visualized.

## Results

### The LC/ESI/MS analysis of *Galaxaura rugosa* tested extracts.

The LC/ESI/MS analysis of the methanol and petroleum ether extracts of *G. rugosa* led to the tentative identification of 57 compounds with their possible fragments. The % identification was 88.15 and 99.00 for methanol and petroleum ether extracts, respectively. The tentatively identified compounds (Table 1) belonged to different phytochemical classes, viz*.* flavonoids, tannins, phenolic acids, phenylpropanoids, alkaloids, triterpenes, etc. It is worth noting that this is the first study evaluating the phytochemical content of *G. rugosa* methanol and petroleum ether extracts collected from Egypt using UPLC/MS. The tentatively identified compounds are summarized in Table 1 and can be detailed as follows.

**Table 1:** Secondary metabolites of the methanol and petroleum ether extracts of *Galaxaura rugosa* identified through tandem mass spectrometry (UPLC/ESI/MS)

No	Compound	Molecular formula	R_t_ (min.)	[M-H]^−^ (*m/z*)	[M + H]^+^/ [M + H + Na]^+^ (*m/z*)	Source (% Composition)		References
						Meth	Pet. Ether	
1	Afzelechin	C_15_H_14_O_5_	0.69	273	275	6.39	33.56	[35]
2	3-methyl-epigallocatechin gallate	C_17_H_34_O_2_	0.77	269	–	0.24	–	[57]
3	Fragment of urolithin A	C_13_H_8_O_4_	0.90	198	–	0.35	–	[42]
4	Fragment	–	1.03	–	132	0.44	–	–
5	Fragment	–	6.74	187	–	0.11	–	–
6	Fragment of vitexin pentoside	–	11.51	293	–	0.27	8.38	[64]
7	Acetyl-*O*-galloyl hexose	C_15_H_19_O_11_	11.66	–	373	0.61	–	[55]
8	Feruloyl-caffeoyl-quinic acid derivative	C_26_H_26_O_12_	12.33	265	–	1.79	23.10	[50]
9	2′′,3′′-dihydro-3′,3′′′-biapigenin methyl ether	C_30_H_20_O_10_	14.11	553	–	1.96	–	[36]
10	Tanshinone V	C_19_H_18_O_3_	14.30	–	316	–	0.99	[59]
11	Fragment	–	14.71	–	304	8.22	–	–
12	Cinnamoyl hexose	C_15_H_18_O_7_	15.00	309	–	–	12.15	[42]
13	Caffeoyl tartaric acid	C_13_H_12_O_9_	15.29	311	–	11.22	-	[42]
14	Chlorogenic acid	C_16_H_18_O_9_	15.57	351	353	–	6.60	[23]
								[54]
15	Isoaloeresin D	C_29_H_32_O_11_	15.60	555	557	4.52	–	[45]
16	Hc4 (dimer)	-	15.94	581	–	1.71	–	[65]
17	3-Sinapoylquinic acid	C_18_H_22_O_10_	16.11	397	–	0.50	3.20	[42]
18	Kaempferol-3-*O*-pentoside	C_21_H_20_O_10_	16.21	441	–	0.24	1.51	[37]
								[39]
								[38]
19	2,3-Didemethyl-(-)-demecolcine	C_19_H_19_NO_5_	16.27	–	344	–	4.57	[63]
20	Menisperine	C_21_H_26_NO_4_	16.41	–	357	0.75	–	[59]
21	Eriodictyol-7-O- hexoside	C_21_H_22_O_11_	16.43	–	451	–	4.06	[40]
22	*p*-Coumaric acid hexoside	C_15_H_18_O_8_	16.58	325	–	7.57	–	[23]
23	Fragment of Caffeic acid derivative	–	16.67	–	332	12.42	-	[55]
24	Kaempferol-*O*-pentose-*O*-hexouronic acid	C_27_H_30_O_17_	16.82	617	–	–	1.82	[23]
25	Aloeresin B	C_29_H_32_O_11_	16.84	393	–	7.24	–	[45]
26	Limocitrol-*O*-hexoside	C_24_H_26_O_14_	17.36	537	–	6.45	–	[41]
27	*p*-Coumaroyl-quinic acid	C_16_H_18_O_8_	17.95	337	–	–	3.58	[51]
28	Caffeoyl-2-hydroxyethane-1,1,2-tricarboxylic acid	–	17.98	339	360	17.72	2.25	[35]
								[52]
29	Fragment of dimeric procyanidin B	–	18.38	407	–	1.76	–	[49]
30	Rosmarinic acid hexoside	C_24_H_26_O_13_	18.94	521	–	1.75	–	[53]
31	Fragment of *cis*-resveratrol-3-*O*-*β*-galloyl-hexoside	C_27_H_26_O_12_	19.26	425	492	1.71	–	[66]
32	Valoneic acid dilactone	C_21_H_10_O_13_	19.56	469	–	0.27	–	[58]
33	Fragment of 13^2^ –Hydroxypheophorbide-*α*-methyl ester	–	20.24	–	485	–	12.34	[53]
34	30 -*O*-Methylcatechin	C_16_H_16_O_6_	20.37	303	305	1.56	–	[42]
35	Fragment of 13^2^ –Hydroxypheophorbide-*α*-methyl ester	–	22.04	–	459	0.41	–	[55]
36	Rhmanocitrin-*O*-coumaroyl hexoside	C_31_H_30_O_14_	22.26	–	609	2.48	–	[43]
37	Caffeic acid 3-*O*-hexouronide	C_15_H_16_O_10_	22.34	355	–	0.49	–	[42]
38	Fragment of sterol ester	–	22.63	381	–	0.39	–	[67]
39	Fragment of Rhmanocitrin- *O*-coumaroyl hexoside	–	22.64	–	475	-	3.72	[43]
40	5-(30,50 -dihydroxyphenyl)- *γ*-valerolactone	C_17_H_20_O_10_	24.90	383	413	0.23	–	[42]
41	3-Hydroxy-12-oleanene-28,29-dioic acid	C_30_ H_46_ O_5_	26.14	486	–	0.07	–	[61]
42	Trimeric ferulic acid	C_30_H_30_O_12_	26.65	685	–	0.11	–	[56]
43	Chrysoeriol-7-*O*-hexouronic acid	C_22_H_20_O_12_	27.03	475	–	0.97	–	[45]
44	Quercetin-7-*O*-hexoside- 3-*O*-(malonyl) hexoside	C_30_H_32_O_20_	27.43	711	–	0.44	–	[44]
45	Salvianolic acid B isomer	C_36_H_30_O_16_	27.86	717	–	0.98	–	[53]
46	Luteolin derivative	–	28.38	–	739	–	1.28	[47]
47	Scutellarein-6-*O*-*β*-D-pentosylhexosyl 7-*O*-*α*-L pentosylhexoside	C_26_ H_28_ O_14_	28.40	564	–	0.43	–	[46]
48	Glycitein 7-*O*-hexouronide	C_22_H_20_O_11_	28.63	459	–	1.24	–	[42]
49	8,11,13-Abietatriene-3,11,12,16-tetrol-12-*O*-*β*-D-hexoside	C_26_H_40_O_9_	28.85	597	–	1.04	–	[60]
50	Propanoic acid, 2-(3-acetoxy-4,4,14-trimethylandrost-8-en-17-yl)	C_27_H_42_O_4_	29.35	–	431	1.38	–	[7]
51	3-Methyl-epigallocatechin gallate	C_23_H_20_O_11_	29.41	471	–	2.84	–	[57]
52	Quercetin-3-*O*-hexouronide	C_21_H_18_O_13_	29.69	477	–	0.54	–	[23]
								[39]
								[49]
53	Hexa-*t*-butylselenatrisiletane	C_24_H_54_SeSi_3_	30.04	505	564	0.95	–	[6]
54	Fragment of trioleoylglycerol	–	30.37	886	888	0.78	–	[67]
55	Aglycone of bidesmosidic triterpene saponin	–	30.50	776	–	0.71	–	[62]
56	Fragment of chlorogenic acid	–	30.61	311	313	0.61	–	[23]
								[54]
57	Taxifolin hexoside	C_21_H_22_O_12_	31.07	465	–	–	2.85	[49]
% Identification								
ESI −ve mode						88.15	99.00	
ESI + ve mode						26.30	26.96	

*Meth.* methanol extract, *Pet. Ether* petroleum ether extract, *Rt* retention time

### Flavonoids

**Fig. 3:**
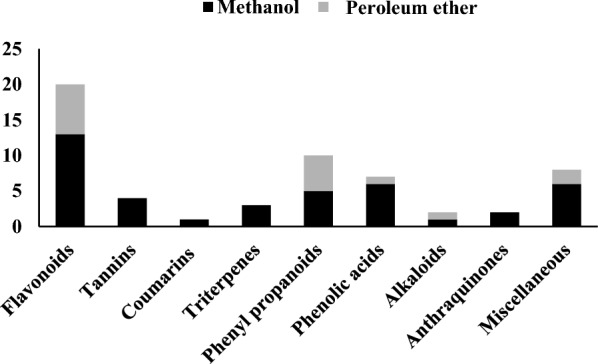
Bar chart showing the main tentatively identified compounds from *Galaxaura rugosa* methanol and petroleum ether extracts

Twenty flavonoids, their glycosides and other derivatives were identified from the methanol and petroleum ether extracts of *G. rugosa* (Table 1) (Fig. 3). A deprotonated molecular ion peak (R_t_ 0.69 min.) was traced at [M-H]^−^ *m/z* 273 and [M + H]^+^ *m/z* 275 and was tentatively identified as afzelechin (6.39% of methanol extract and 33.56% of petroleum ether extract) [35]. An apigenin biflavonoid was identified (R_t_ 14.11 min.) at [M-H]^−^ *m/z* 553 and was for 2′′,3′′-dihydro-3′,3′′′-biapigenin methyl ether [36]. Kaempferol-3-*O*-pentoside, a well-known and common flavonoid glycoside, showed a deprotonated molecular ion peak (R_t_ 16.21 min.) at [M-H]^−^ *m/z* 441 [37–39]. Another flavonoid glycoside was traced at (R_t_ 16.43 min.) [M + H]^+^ *m/z* 451 [40]. Similarly, another kaempferol derivative had a molecular ion peak at (R_t_ 16.82 min.) [M-H]^−^ *m/z* 617 and was tentatively identified as kaempferol-*O*-pentose-*O*-hexouronic acid [23]. The presence of a pseudomolecular ion peak at (R_t_ 17.36 min.) [M-H]^−^ *m/z* 537 allowed for the identification of limocitrol-*O*-hexoside (6.45% of the methanol extract) [41].

One flavonol was identified from the methanol extract, and it recorded a molecular ion peak at [M-H]^−^ *m/z* 303 and [M + H]^+^ *m/z* 305, and it was assigned to 30 -*O*-methylcatechin [42]. Another flavonoid glycoside with an attached phenolic acid group was identified as rhmanocitrin-*O*-coumaroyl hexoside, and its identity was shown by the existence of a molecular ion peak at *m/z* 609 in positive mode [43] also, this same coumaroyl flavonoid glycoside showed one of its fragments at *m/z* 475 in positive mode [43]. In the same context, quercetin-7-*O*-hexoside- 3-*O*-(malonyl) hexoside was traced at [M-H]^−^ *m/z* 711 [44], while Chrysoeriol-7-*O*-hexouronic acid was tentatively identified at [M-H]^−^ *m/z* 475 [45]. In addition to that, scutellarein-6-*O*-*β*-D-pentosylhexosyl 7-*O*-*α*-L-pentosylhexoside was recorded at [M-H]^−^ *m/z* 564 [46], luteolin derivative at [M + H]^+^ *m/z* 739 [47], quercetin-3-*O*-hexouronide at [M-H]^−^ *m/z* 477 [23, 39, 48] together with taxifolin hexoside at [M-H]^−^ *m/z* 465 [49]. One isoflavonoid was recorded at [M-H]^−^ *m/z* 459 for glycitein-7-*O*-hexouronide [42].

### Phenyl propanoids

Phenyl propanoids represented the second most abundant class identified from *G. rugosa* methanol and petroleum ether extracts (Table 1) (Fig. 3). Ten phenyl propanoids and their derivatives were tentatively identified from the two extracts and can be detailed as follows; compound 8 showed a molecular ion peak at [M-H]^−^ *m/z* 265 and was tentatively identified as feruloyl-caffeoyl-quinic acid derivative [50], and was one of the major compounds in the petroleum ether extract (23.10%). Another quinic acid derivative was traced at [M-H]^−^ *m/z* 397 and was found to be 3-sinapoylquinic acid [42]. Similarly, *p*-coumaroyl-quinic acid showed a molecular ion peak at *m/z* 337 [51], and was found only in the petroleum ether extract (3.58%). Compound 28 was assigned to be one of the caffeic acid derivatives with a molecular ion peak at *m/z* 339 in negative mode and *m/z* 360 in positive mode due to ammonium adduct [M + H + NH_4_]^+^ [35, 52]. One glycoside derivative of phenyl propanoid was recorded at [M-H]^−^ *m/z* 521 and was tentatively assigned to rosmarinic acid hexoside [53]. Compounds 14 with [M-H]^−^ *m/z* 351 and [M + H]^+^ *m/z* 353 were tentatively recorded for chlorogenic acid (6.60% of the petroleum ether extract), which showed one of its fragments at [M-H]^−^ *m/z* 311 and [M + H]^+^ *m/z* 313 [23, 54].

### Phenolic acids

Six phenolic acids were traced from the methanol extract in addition to only one from the petroleum ether extract of *G. rugosa* (Table 1) (Fig. 3). Compound 7 was traced at [M + H]^+^ *m/z* 373 with a molecular formula of C_15_H_19_O_11_ and was tentatively assigned to acetyl-*O*-galloyl hexose [55]. Another hexoside derivative was shown at *m/z* 309 in ESI negative mode and was identified as cinnamoyl hexose (only in the petroleum ether extract, 12.15%) [42]. Compound 13 showed a molecular ion peak at [M-H]^−^ *m/z* 311 and a molecular formula of C_13_H_12_O_9_ and was recorded to be caffeoyl tartaric acid (11.22% of the methanol extract) [42]. Similarly, compound 22 with [M-H]^−^ *m/z* 325 was tentatively identified as *p*-coumaric acid hexoside (7.57% of the methanol extract) [23]. Another glycoside derivative of a phenolic acid was spotted at m/z 355 in ESI negative mode and a molecular formula of C_15_H_16_O_10_ and was assigned to caffeic acid 3-*O*-hexouronide [42]. Another phenolic acid derivative was tentatively identified at *m/z* 685 (ESI negative) and was linked to the presence of trimeric ferulic acid [56].

### Tannins

Four tannins and tannin derivatives of both the hydrolyzable and the condensed types were only identified from the methanol extract of *G. rugosa* (Table 1) (Fig. 3). Compound 2 with a molecular ion peak at [M-H]^−^ *m/z* 269 and a molecular formula of C_17_H_34_O_2_ was identified as 3-methyl-epigallocatechin gallate [57]. Similarly, 3-methyl-epigallocatechin gallate showed a peak at *m/z* 471 in ESI negative mode [57]. A fragment of dimeric procyanidin B showed a peak at *m/z* 407 in the negative ion mode [49]. Compound 32 showed a molecular ion peak at [M-H]^−^ *m/z* 469 and was tentatively assigned to the hydrolyzable tannin valoneic acid dilactone [58].

### Diterpenes

One diterpene was tentatively identified as compound 10 (Table 1) from the petroleum ether extract, and it showed a molecular ion peak at [M + H]^+^ *m/z* 316 and was reported to be tanshinone V [59]. Another diterpene was assigned to 8,11,13-abietatriene-3,11,12,16-tetrol-12-*O*-*β*-D-hexoside with *m/z* 597 in ESI negative mode (methanol extract only) [60].

### Triterpenes

Two diterpenes were traced from the extracts of G. rugosa and three other triterpenes (Table 1 and Fig. 3). The three identified triterpenes were only traced from the methanol extract (compounds 41, 50, and 55, Table 1). Compound 41 was represented with a molecular ion peak at [M-H]^−^ *m/z* 486 for 3-hydroxy-12-oleanene-28,29-dioic acid [61], while compound 50 showed its peak in the positive ion mode at *m/z* 431 and was assigned to propanoic acid, 2-(3-acetoxy-4,4,14-trimethylandrost-8-en-17-yl). In addition to that, compound 51 was tentatively identified at *m/z* 776 for the aglycone of bidesmosidic triterpene saponin [62].

### Alkaloids

Two alkaloids were recorded from the extracts of *G. rugosa* (Table 1) (Fig. 3). In the ESI positive mode, compound 19 showed a molecular ion peak at *m/z* 344 and was tentatively identified as 2,3-dimethyl-(-)-demecolcine (4.57% of the petroleum ether extract) [63]. Compound 20 had a molecular ion peak at [M + H]^+^ *m/z* 357 (methanol extract only) and was assigned to menisperine [59].

### The activity of *Galaxaura rugosa* tested extracts against *Anopheles pharoensis*

**Fig. 4:**
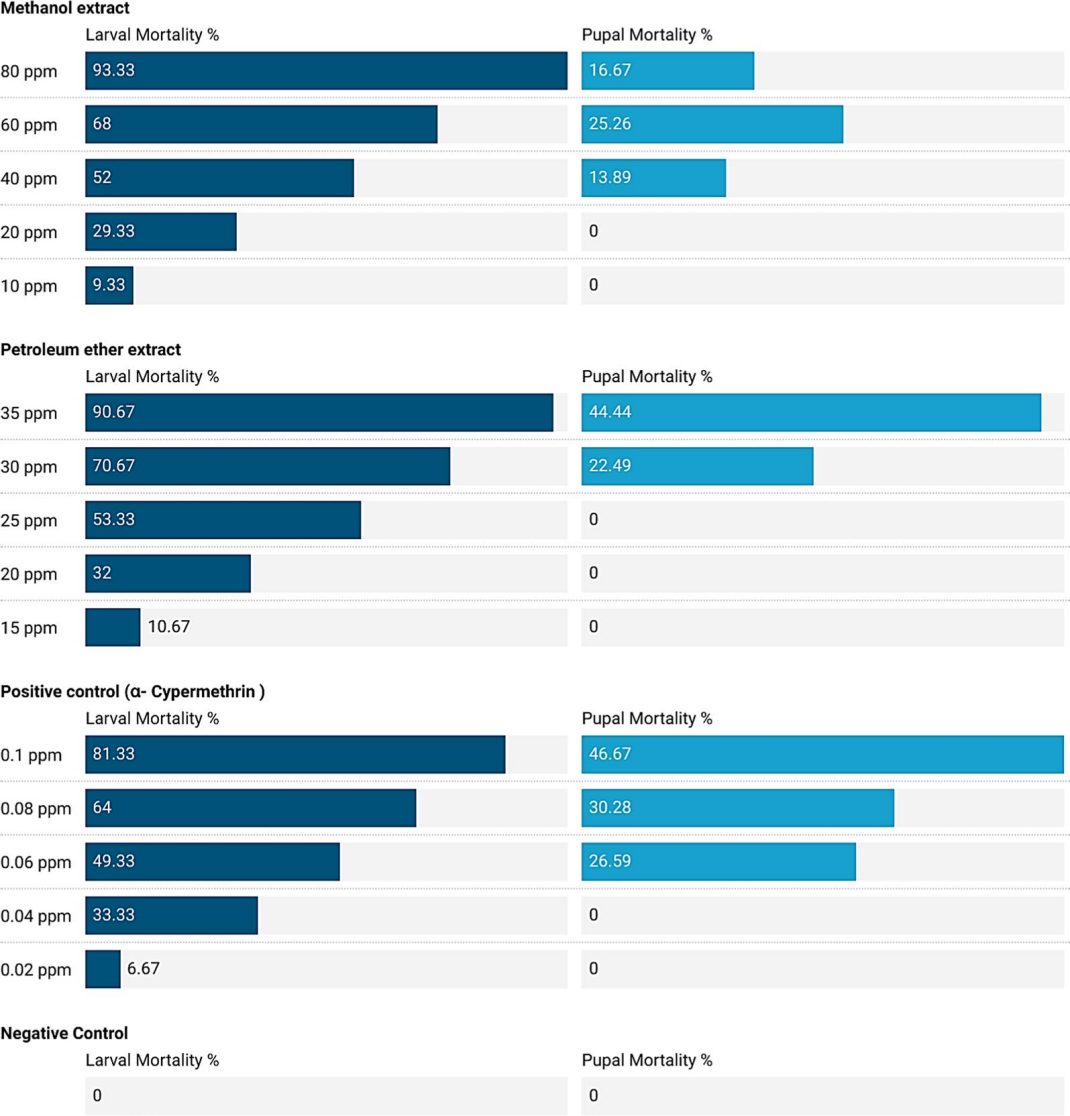
Larval and pupal mortality of *Anopheles pharoensis* induced by methanol and petroleum ether extracts of *Galaxaura rugosa*

The highest larval mortality (93.33 and 90.67%) was recorded at the highest concentrations (80 and 35 ppm) of *G. rugosa* methanol and petroleum ether extracts, respectively in compared to 81.33% mortality at 0.1 ppm for positive control. Meanwhile, the lowest larval mortality values (9.33 and 10.67%) were achieved by the lowest concentrations (10 and 15 ppm), respectively when compared with 0.0% for the control group. Also, pupal mortality was recorded at 44.44 and 22.48% at 35 and 30 ppm of *G. rugosa* petroleum ether extract, respectively, compared with 0.0% for the control group (Additional file 1: Table S1, and Fig. 4).

Methanol and petroleum extract varied significantly (P < 0.05) in comparison to the negative control but didn’t vary differently with the positive control (P > 0.05) with regard to larval mortality and adult emergency. While methanol extract didn’t vary statistically with the positive control (P > 0.05), petroleum ether extract varied with the positive control (P < 0.05) regarding pupal mortality. It has been observed that, as concentration increases, more significant (P < 0.05) mortality increases and the opposite for adult emergency (Fig. 4).

Methanol and petroleum ether extracts of *G. rugosa* prolonged larval and pupal durations at all concentrations. The larval duration was prolonged from 4.19 days in the control groups to 5.31 and 5.64 days for the methanol and petroleum ether extracts at 80 and 35 ppm, respectively. Both extracts showed a suppressive impact on the growth index throughout the board. Growth index recorded 6.05 and 8.75 by 35 and 30 ppm of petroleum ether extract, compared with 15.6 for the control congers **(**Additional file 1: Table S1).

Statistically, positive control varied significantly (P < 0.05) with the methanol extract and did not differ (P > 0.05) with the petroleum ether extract regarding larval, pupal duration, developmental times, and growth index. Concentration has a significant effect (P < 0.05) on larval and pupal duration as developmental time is required and the growth index observed for insects (Additional file 1: Table S1).

**Fig. 5:**
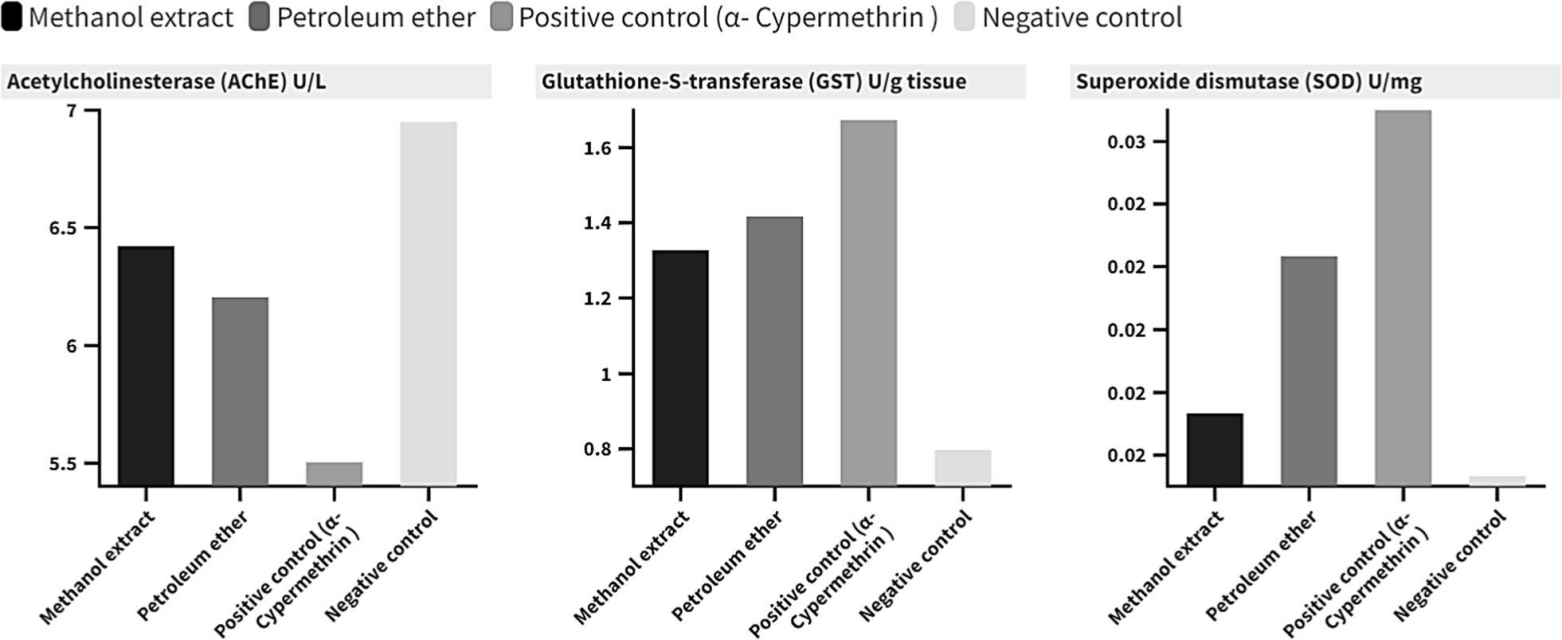
Gradient column chart represents the effect of *Galaxaura rugosa* methanol and petroleum ether extracts on Acetylcholinesterase (AChE), Glutathione-S-transferase (GST), and Superoxide dismutase (SOD) activity in 3rd instar larvae of *Anopheles pharoensis*

In addition, the tested methanol and petroleum ether extracts decreased the AChE activity of 3rd instar larvae of *An. pharoensis*, as it recorded 6.42 and 6.20 U/L, compared with 6.95 U/L for the untreated group. The tested extracts promoted GST activity, increasing from 0.79 U/g tissue for the control group to 1.32 and 1.41 U/g tissue for the methanol and petroleum ether extracts, respectively (Additional file 1: Table S2 and Fig. 5). Overall, both extracts had significantly (P < 0.05) affected the studied enzyme in comparison to positive control. The same was observed with negative control, except methanol extract, which did not affect Superoxide dismutase (SOD) U/mg normal levels (Fig. 5).

**Fig. 6:**
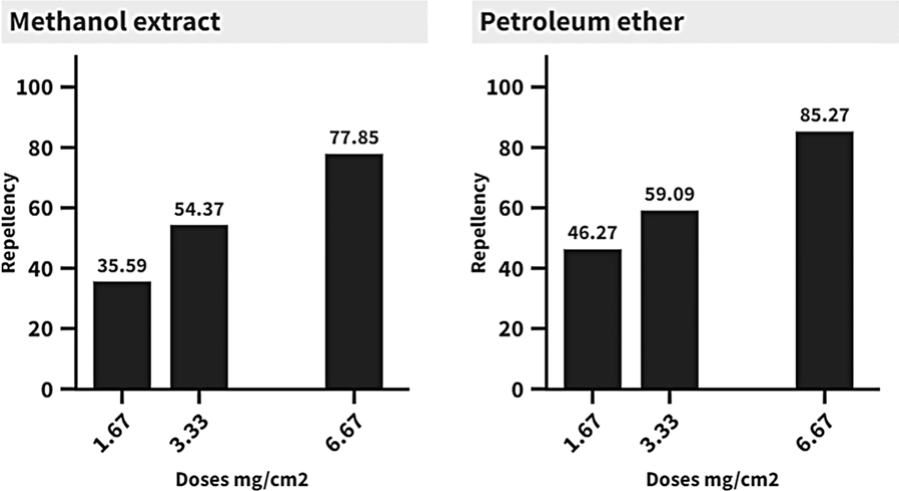
Gradient column chart of repellent activity of *Galaxaura rugosa* methanol and petroleum ether extracts against *Anopheles pharoensis* starved females

Also, the petroleum ether extract of* G. rugosa* recorded the highest repellent activity (85.26%) at 6.67 mg/cm^2^, respectively; meanwhile, the methanol extract provided 77.85% protection from *An. pharoensis* females bites at the same dose, in comparison to 100.0% protection recorded by the positive control (DEET) at 1.8 mg/cm^2^, respectively. Statistically, positive control varied significantly (P < 0.05) with both extracts (Additional file 1: Table S3 and Fig. 6).

### Effect on non-target organisms

Zebrafish *(*and *Daphnia magna* were the two non-target organisms’ models used for this research. Obtained results showed that LC_25_ values were 829.5 and 377.4 µg/mL against Zebrafish for methanol and petroleum ether extracts of *G. rugosa,* while the LC_50_ values were 1988.8 and 1365.1 µg/mL, respectively. Also, LC_75_ values recorded 4768.7 and 4937.4 µg/mL by *G. rugosa* methanol and petroleum ether extracts against Zebrafish, respectively (Table 2).

**Table 2:** LC_25_, LC_50,_ and LC_75_ of *Galaxaura rugosa* methanol and petroleum ether extracts against Zebrafish and *Daphnia magna* after 48 h of exposure

Organism	Extract	LC_25_ (LCL-UCL)	LC_50_ (LCL-UCL)	LC_75_ (LCL-UCL)
Zebrafish *(Danio rerio)*	Methanol extract	829.5 (530.6–2927.7)	1988.8 (1009.1–26320.7)	829.5 (530.6–2927.7)
	Petroleum ether extract	377.4 (233.3–752.6)	1365.1 (702.6–8566.7)	377.4 (233.3–752.6)
	α-Cypermethrin	0.475 (0.422–0.541)	0.413 (0.388–0.465)	0.383 (0.349–0.426)
*Daphnia magna*	Methanol extract	8.25 (5.72–10.18)	11.65 (9.25–13.89)	8.25 (5.72–10.18)
	Petroleum ether extract	10.09 (8.96–11.08)	14.36 (13.28–15.44)	10.09 (8.96–11.08)
	α-Cypermethrin	0.004 (0.003–0.008)	0.007 (0.005–0.011)	0.010 (0.008–0.014)

*LCL* Lower 95%Confidential Limit, *UCL* Upper 95% Confidential Limit. All values are represented by µg/mL

On the other hand, LC_25_ values of 8.25 and 10.09 µg/mL against *Daphnia magna* were recorded by the methanol extract and the petroleum ether extracts, respectively while LC_50_ values were 11.65 and 14.36 µg/mL, respectively. Finally, LC_75_ values were 16.44 and 20.45 µg/mL against *Daphnia* after 48 h of the exposure recorded by *G. rugosa* methanol extract and petroleum ether extracts, respectively (Table 3).

**Table 3:** Effect of *Galaxaura rugosa* methanol and petroleum ether extracts on different developmental times of *Anopheles pharoensis*

Extract	Conc (ppm)	Larval duration (Days ± SD)	Pupal duration (Days ± SD)	Developmental time (Days ± SD)	Growth index
Methanol extract	80	5.31 ± 0.032^a^	3.32 ± 0.028^a^	8.64 ± 0.032^a^	9.64 ± 2.72^bc^
	60	5.10 ± 0.024^b^	3.25 ± 0.029^a^	8.35 ± 0.036^b^	8.94 ± 0.27^c^
	40	4.77 ± 0.096^c^	3.07 ± 0.029^b^	7.84 ± 0.125^c^	10.98 ± 0.56^abc^
	20	4.54 ± 0.061^d^	2.76 ± 0.037^c^	7.30 ± 0.061^d^	13.69 ± 0.11^ab^
	10	4.47 ± 0.045^d^	2.45 ± 0.044^d^	6.92 ± 0.09^e^	14.43 ± 0.18^a^
Petroleum ether extract	35	5.64 ± 0.047^a^	3.52 ± 0.049^a^	9.17 ± 0.096^a^	6.05 ± 0.79^c^
	30	5.44 ± 0.046^b^	3.41 ± 0.012^a^	8.86 ± 0.042^b^	8.75 ± 0.58^b^
	25	5.26 ± 0.037^c^	3.35 ± 0.016^ab^	8.61 ± 0.049^b^	11.61 ± 0.06^a^
	20	5.11 ± 0.021^c^	3.22 ± 0.072^b^	8.33 ± 0.084^c^	12.0 ± 0.12^a^
	15	4.93 ± 0.074^a^	3.02 ± 0.087^c^	7.95 ± 0.089^d^	12.56 ± 0.14^a^
Positive control (α- Cypermethrin)	0.1	5.77 ± 0.082^a^	3.67 ± 0.054^a^	9.45 ± 0.106^a^	5.66 ± 1.57^b^
	0.08	5.41 ± 0.153^b^	3.63 ± 0.032^ab^	9.04 ± 0.179^b^	7.69 ± 0.67^b^
	0.06	5.35 ± 0.062^b^	3.53 ± 0.021^bc^	8.88 ± 0.057^bc^	8.25 ± 0.5^b^
	0.04	5.19 ± 0.036^b^	3.45 ± 0.032^ cd^	8.65 ± 0.037^ cd^	11.56 ± 0.04^a^
	0.02	5.15 ± 0.028^b^	3.34 ± 0.036^d^	8.50 ± 0.065^d^	11.76 ± 0.09^a^
Negative Control		4.19 ± 0.131	2.21 ± 0.044	6.41 ± 0.086	15.6 ± 0.21

Means that do not share a letter are significantly different. Growth index has been calculated according to Shehata et al. [12]

**Fig. 7:**
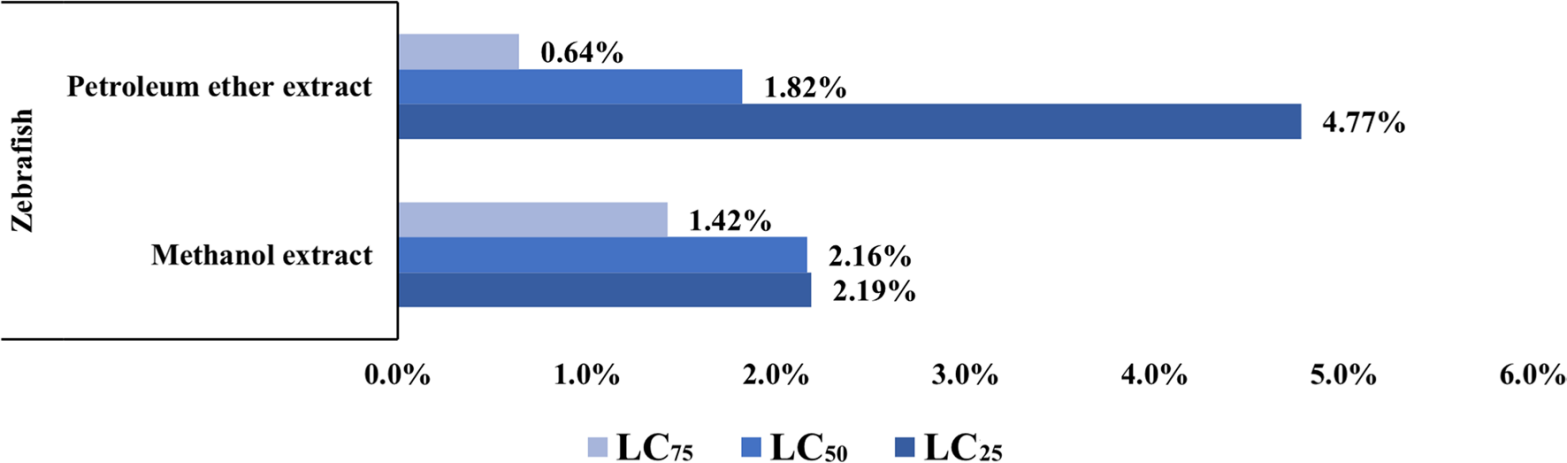
Comparison between lethal concentration values of methanol and petroleum ether extracts against mosquito larvae and the non-target models (Zebrafish and *Daphnia magna*)

**Fig. 8:**
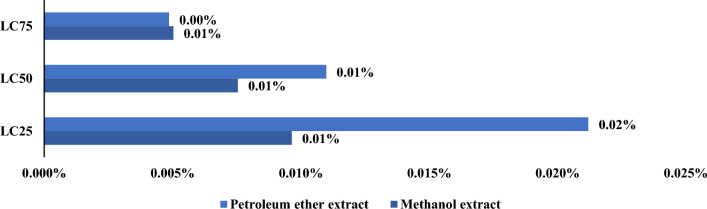
Comparison between lethal concentration values of methanol and petroleum ether extracts against α-cypermethrin on non-target models (Zebrafish)

**Fig. 9:**
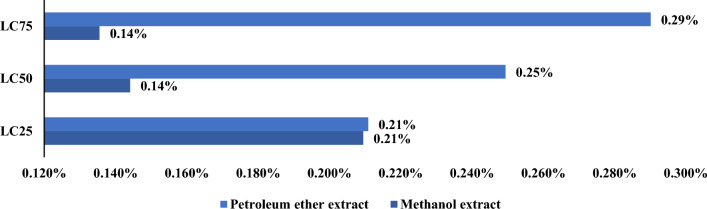
Comparison between lethal concentration values of methanol and petroleum ether extracts against α-cypermethrin on non-target models (*Daphnia magna*)

The ratio of the Zebrafish toxicity values to the mosquito larvae toxicity values for the investigated extracts was statistically significant. The concentration values compared at the LC_25_ level were 18.4 and 829.5 (folds, percent change); at the LC_50_ level, they were 43.03 and 1988.8, and at the LC_75_ level, they were 67.9 and 4768.7 (folds, percent change), and at the LC_75_ level, they were 31.71 and 4947.4. (Mosquito larva: zebrafish). These findings corroborate the low toxicity of the investigated compounds against mosquito larvae, suggesting that their toxicity to other organisms was similarly low (Figs. 7, 8, 9).

## Discussion

Red algae represent a biologically important part of marine life; they carry many phytoconstituents with potent biological activity. The Genus *Galaxaura* is chemically under-studied, and scarce scientific literature was traced concerning the phytoconstituents of its members. In this study, the methanol and petroleum ether extracts of *G. rugosa* were analysed through UPLC/ESI/MS, and 57 secondary metabolites were identified and quantified, as discussed before in the results section. Flavonoids were the most abundant class, followed by phenyl propanoids, phenolic acids, and tannins. When comparing the two *G. rugosa* extracts, the methanol extract was richer in flavonoids, tannins, coumarins, and phenolic acids than the petroleum ether extract, while both showed the same number of identified phenyl propanoids. The extract of *G. rugosa* showed antioxidant (IC_50_ = 81.00 μg GAE/ml), antityrosinase and antielastase activities (IC_50_ = 88.00 μg GAE/ml and IC_50_ = 243.00 μg GAE/ml, respectively) [64–68]. Silver nanoparticles prepared from *G. rugosa* methanol extract had antibacterial activity against multidrug-resistant bacteria [69]. Moreover, chloroform extract of *G. rugosa* had antibacterial activity against *Klebsiella pneumoniae* (24 mm, 0.15 mg/ml) and antifungal activity against *Aspergillus fumigatus*, *Aspergillus niger* and *Candida tropicalis* with (inhibition zones of 21, 22, and 25 mm, IC_50_ = 1.25, 0.312, and 0.156 mg/ml), respectively. The extract also showed both antioxidant (80.96%, IC_50_ = 27.8 μg/ml) and cytotoxic activities (IC_50_ = 15 ± 1.7) [6]. In addition to that, the dichloromethane (DCM) extract of *G. rugosa* was phytochemically evaluated and tested for an inflammation model in rats (ear edema model). The DCM extract was rich in fatty acids, steroids, tritepenoids, and carbohydrates; besides, it displayed potent anti-inflammatory activity by reducing writhing (> 75% at the dose of 6 mg/kg) [70]. The metabolic profiling of other red algae belonging to genus *Galaxaura *viz*. G. elongata* was reported in the literature. The analysis was accomplished through GC/MS, where *G. elongata* methanol extract was rich in flavonoids, steroids, terpenoids, saponins, tannins, and phenols. The main identified compounds were 3R*,4S*-3-(2-nitro-4-methoxy phenyl)-4-(4-hydroxy phenyl) hexane (7.97%), cyclopropane nonanoic acid, methyl ester (2.29%) and di isooctylphthalate (2.25%). The red algae extract showed potent antimicrobial activity against *Candida albicans* (16.07 ± 0.21 mm, inhibition zone) [6].

Based on the solvent utilized for extraction and the strength of the extract, the current investigation found that *G. rugosa* extracts exhibited particularly effective larvicidal activity against *An. pharoensis* third instar. The LC_50_ results indicated that petroleum ether extract was more effective than methanol extract against the test larvae. Extracts evaluated at all concentrations were also shown to increase the length of both the larval and pupal stages. It has been hypothesized that triterpenes components contribute to the larvicidal action of the studied extracts [71, 72]. Recorded larvicidal activity confirms the previous findings where chloroform and methanol extracts of seaweed, *Bryopsis pennata,* recorded larvicidal activity (LC_50_ = 82.55 and 160.07 mg/mL) against *Aedes aegypti* larvae, as well as inducing a strong prolongation in larval period (1.5-fold longer than control) [73]; ethyl acetate extract of *Caulerpa racemosa* exhibited larvicidal activity against *Ae. aegypti* with LC_50_ and LC_90_ values of 579.9, 1255.4 and 495.4, 1073.9 ppm at 24 and 48 h, respectively [74]; methanol crude extract of *Halymenia palmata* and its fractions (Hpf-1 and Hpf-2) induced mortality in *Ae. aegypti* larvae with LC_50_ and LC_90_ values of 42.73 and 95.48 μg/mL for crude extract; 91.95 and 709.04 μg/mL for Hpf-1; 23.69 and 233.49 μg/mL for Hpf-2, respectively [75]; ethanolic extracts of *Chaetomorpha linum, Ulva intestinalis,* and *Sargassum dentifolium* algae showed larvicidal activity against *Culex pipiens* 3rd instar with LC_50_ equal to 224.45, 231.06 and 241.79 ppm at 48 h exposure, respectively [76].

Also, a depression in acetylcholinesterase (AChE) level in *An. pharoensis* third larval instar was recorded. As a biomarker of exposure to certain classes of pollutants, AChE activity measurements have become commonplace [77]. On the other hand, an elevated glutathione-S-transferase (GST) level in *An. pharoensis* larvae was recorded by the tested extracts; Biotransformation of foreign chemicals, drug metabolism, and protection from oxidative damage are all aided by GST [78]. While superoxide dismutase (SOD) of *An. pharoensis* larvae, a major component of mosquitoes’ antioxidant defense system [79], was not affected by tested extracts, respectively. Generally, the effect of *G. rugosa* methanol and petroleum ether extracts on AChE, GST, and SOD confirmd the results recorded using different plant extracts against *Cx. pipiens* larvae [80, 81].

A correlation was also found between the extract's repellent properties, the solvent it was extracted with, and the amount of extract utilized. The complexity of the chemical makeup of the examined extracts' components is reflected in their repellant action [82]. All concentrations of *G. rugosa* extracts effectively deter female* An. pharoensis* from feeding on their dead. Repellant activity measured varied with dosage and extraction solvent. In general, the repellent efficacy of petroleum ether extract was greater against *An. pharoensis* starving females than methanol extracts. The repellent activity of tested extracts can be due to the presence of phenolic acids, terpenoids, and alkaloids, which exist in the tested extracts; these compounds may jointly or independently contribute to producing a repellent activity [83]. The repellent activity of the tested extracts was consistent with that reported using different plant extracts against *Cx. pipiens, Ae. aegypti, Anopheles stephensi, Culex quinquefasciatus,* and *An. pharoensis* starved females [10, 84, 85].

Zebrafish are useful for studying natural insecticides because they share some biological and ecological features with mosquitoes, such as being aquatic, diurnal, and having a short life cycle.

Zebrafish, a sensitive non-target organism bioindicator, and *Daphnia magna*, a highly important environmental bioindicator, show no signs of toxicity to the extracted components. The same results were previously recorded, as isolated compounds derived from the stem bark of Annickia chlorantha showed mosquitocidal activity against *Cx. pipiens* and did not cause significant mortality or malformations in Zebrafish, indicating their safety for non-target organisms [86]. *Daphnia* is sensitive to various natural and synthetic insecticides [87]. The acute toxicity to daphnids varied less than tenfold across seven alkaloids compared with crude plant extracts [88].

## Conclusion

*Galaxaura rugosa* was studied for its action against the malarial vector *Anopheles pharoensis* and non-target species *Danio rerio* and *Daphnia magna* and its UPLC/ESI/MS profile using methanol and petroleum ether extracts. In addition, further research is required to clarify whether or if *G. rugosa* is effective against mosquitoes of other species. However, research into the separated chemicals' insecticidal action should accompany the extracts' in-depth isolation and structural elucidation. Finally, replacing synthetic pesticides with compounds from red algae for mosquito control may have less of an impact on the environment and save money.

### Supplementary Information

**Additional file 1: Table S1. **Toxicity of *Galaxaura rugosa *methanol and petroleum ether extracts on *Anopheles pharoensis* immature stages.** Table S2.** Effect of *Galaxaura rugosa *methanol and petroleum ether extracts on Acetylcholinesterase (AChE), Glutathione-S-transferase (GST), and Superoxide dismutase (SOD) activity in 3^rd^ instar larvae of *Anopheles pharoensis.*** Table S3.** Repellent activity of *Galaxaura rugosa *methanol and petroleum ether extracts against *Anopheles pharoensis *starved females.

## Data Availability

The datasets used and/or analyzed during the current study are available from the corresponding author on reasonable request.

## Abbreviations

AChE: Acetylcholinesterase
An.: Anopheles
Cx.: Culex
G.: Galaxaura
GST: Glutathione S-transferase
SOD: Superoxide dismutase
