# Supplementary material for: UPLC/ESI/MS profiling of red algae Galaxaura rugosa extracts and its activity against malaria mosquito vector, Anopheles pharoensis, with reference to Danio rerio and Daphnia magna as bioindicators
Source: Malar J. 2023 Dec 1;22:368. doi: 10.1186/s12936-023-04795-w (PMC10691061; doi:10.1186/s12936-023-04795-w)
Supplement: Supplementary file 1 — Additional file 1: Table S1. Toxicity of Galaxaura rugosa methanol and petroleum ether extracts on Anopheles pharoensis immature stages. Table S2. Effect of Galaxaura rugosa methanol and petroleum ether extracts on Acetylcholinesterase (AChE), Glutathione-S-transferase (GST), and Superoxide dismutase (SOD) activity in 3rd instar larvae of Anopheles pharoensis. Table S3. Repellent activity of Galaxaura rugosa methanol and petroleum ether extracts against Anopheles pharoensis starved females. [file 12936_2023_4795_MOESM1_ESM.docx]

**UPLC/ESI/MS profiling of red algae *Galaxaura rugosa* extracts and its activity against malaria mosquito vector, *Anopheles pharoensis*, with reference to *Danio rerio* and *Daphnia magna* as bioindicators**

Mohamed A.M. El-Tabakh^1*^, Esraa A. Elhawary^2^, Hossam M. Hwihy^1^, Kareem F. Darweesh^1^, Raafat M. Shaapan^3^, Emad A. Ghazala^4^, Mostafa M. Mokhtar^1^, Hassan O. Waheeb^1^, Deyaa E.M. Emam^1^, Nader A. Bakr^1^, Ahmed Z.I. Shehata^1^

^1^Zoology Department, Faculty of Science, Al-Azhar University, Cairo 11651, Egypt

^2^Department of Pharmacognosy, Faculty of Pharmacy, Ain-Shams University, Cairo, Egypt

^3^Department of zoonosis, Veterinary Research Institute, National Research Centre, Giza, Egypt

^4^EEAA, Ras Muhammed National Park, South Sinai, Egypt

*Corresponding author

Email: [dr.m.eltabakh.201@azhar.edu.eg](mailto:dr.m.eltabakh.201@azhar.edu.eg)

**Additional file 1: Table S1.** Toxicity of *Galaxaura rugosa* methanol and petroleum ether extracts on *Anopheles pharoensis* immature stages.

| Extract | Conc.  (ppm) | Larval Mortality  (%) | Pupal Mortality  (%) | Adult Emergence  (%) |
| --- | --- | --- | --- | --- |
| Methanol extract | 80 | 93.33±1.88^a^ | 16.67±23.57^b^ | 83.33±23.57^a^ |
|  | 60 | 68.0±3.26^b^ | 25.26±2.59^a^ | 74.73±2.59^b^ |
|  | 40 | 52.0±0.0^c^ | 13.89±3.93^b^ | 86.11±3.93^c^ |
|  | 20 | 29.33±1.88^d^ | 0.0^a^ | 100.0±0.0^d^ |
|  | 10 | 9.33±3.77^e^ | 0.0^b^ | 100.0±0.0^e^ |
| Petroleum ether extract | 35 | 90.67±1.88^a^ | 44.44±7.85^a^ | 55.55±7.85^a^ |
|  | 30 | 70.67±4.98^b^ | 22.48±4.86^a^ | 77.51±4.86b |
|  | 25 | 53.33±3.77^c^ | 0.0^b^ | 100.0±0.0^c^ |
|  | 20 | 32.0±3.26^d^ | 0.0^b^ | 100.0±0.0^d^ |
|  | 15 | 10.67±3.77^e^ | 0.0^b^ | 100.0±0.0^e^ |
| Positive control  (α- Cypermethrin) | 0.1 | 81.33±4.98^a^ | 46.67±14.4^b^ | 53.33±14.4^d^ |
|  | 0.08 | 64.0±3.26^b^ | 30.27±7.46^ab^ | 69.72±7.46^c^ |
|  | 0.06 | 49.33±3.77^c^ | 26.58±4.98^a^ | 73.41±4.98^c^ |
|  | 0.04 | 33.33±1.88^d^ | 0.0^c^ | 100.0±0.0^b^ |
|  | 0.02 | 6.67±1.88^e^ | 0.0^c^ | 100.0±0.0^a^ |
| Negative Control | | 0.0 | 0.0 | 100.0±0.0 |

Means that do not share a letter are significantly different.

**Additional file 1: Table S2.** Effect of *Galaxaura rugosa* methanol and petroleum ether extracts on Acetylcholinesterase (AChE), Glutathione-S-transferase (GST), and Superoxide dismutase (SOD) activity in 3^rd^ instar larvae of *Anopheles pharoensis.*

| Extract | Acetylcholinesterase  (AChE)  U/L | Glutathione-S-transferase  (GST)  U/g tissue | Superoxide dismutase  (SOD)  U/mg |
| --- | --- | --- | --- |
| Methanol extract | 6.42±0.014^a^ | 1.32±0.03^a^ | 0.01±0.0012^c^ |
| Petroleum ether | 6.20±0.012^b^ | 1.41±0.02^b^ | 0.02±0.0012^b^ |
| Positive control  (α- Cypermethrin) | 5.50±0.009^c^ | 1.67±0.012^c^ | 0.02±0.0008^a^ |
| Negative Control | 6.95±0.114^d^ | 0.79±0.012^d^ | 0.01±0.0009^c^ |

Means that do not share a letter are significantly different.

**Additional file 1: Table S3.** Repellent activity of *Galaxaura rugosa* methanol and petroleum ether extracts against *Anopheles pharoensis* starved females.

| Extract | Doses  mg/cm^2^ | Repellency % |
| --- | --- | --- |
| Methanol extract^b^ | 6.67 | 77.85±3.27^a^ |
|  | 3.33 | 54.36±0.51^b^ |
|  | 1.67 | 35.59±2.78^c^ |
| Petroleum ether extract^b^ | 6.67 | 85.26±4.03^a^ |
|  | 3.33 | 59.09±3.06^b^ |
|  | 1.67 | 46.27±3.94^c^ |
| Positive Control (DEET)^a^ | 1.8 | 100.0±0.0 |
| Negative control^c^ | | 0.0 |

Means that do not share a letter are significantly different.
